# Cross-Contamination Quantification in Powders for Additive Manufacturing: A Study on Ti-6Al-4V and Maraging Steel

**DOI:** 10.3390/ma12152342

**Published:** 2019-07-24

**Authors:** Eleonora Santecchia, Paolo Mengucci, Andrea Gatto, Elena Bassoli, Silvio Defanti, Gianni Barucca

**Affiliations:** 1 Consorzio Interuniversitario Nazionale per la Scienza e Tecnologia dei Materiali (INSTM-UdR Ancona), Via Brecce Bianche 12, 60131 Ancona, Italy; 2 Dipartimento SIMAU, Università Politecnica delle Marche, Via Brecce Bianche 12, 60131 Ancona, Italy; 3 Università di Modena e Reggio Emilia, Dipartimento DIEF, Via Vivarelli 10, 41125 Modena, Italy

**Keywords:** PBF, cross-contamination, Ti-6Al-4V, maraging steel

## Abstract

Metal additive manufacturing is now taking the lead over traditional manufacturing techniques in applications such as aerospace and biomedicine, which are characterized by low production volumes and high levels of customization. While fulfilling these requirements is the strength of metal additive manufacturing, respecting the tight tolerances typical of the mentioned applications is a harder task to accomplish. Powder bed fusion (PBF) is a class of additive manufacturing in which layers of metal powder are fused on top of each other by a high-energy beam (laser or electron beam) according to a computer-aided design (CAD) model. The quality of raw powders for PBF affects the mechanical properties of additively manufactured parts strongly, and therefore it is crucial to avoid the presence of any source of contamination, particularly cross-contamination. In this study, the identification and quantification of cross-contamination in powders of Ti-6Al-4V and maraging steel was performed using scanning electron microscopy (SEM) and energy-dispersive spectroscopy (EDS) techniques. Experimental results showed an overall good reliability of the developed method, opening the way for applications in machine learning environments.

## 1. Introduction

Manufacturing complex shapes without additional costs is certainly one of the outstanding advantages of metal additive manufacturing. This range of techniques also allows a high freedom of design with consequent savings in materials and weight. One of the most widely used additive manufacturing techniques for metals and alloys is laser powder bed fusion (PBF), during which layers of powder feedstock are selectively fused one on top of the other by a high-energy laser, while unfused powder supports the manufactured part [1]. More than fifty process parameters can be finely tuned to customize the microstructure of the used metallic material and the mechanical behavior of the final part [2,3]. However, while machine working parameters can be fixed quite reliably, one of the keys to ensuring repeatability of the laser powder bed fusion process is the quality of the powder feedstock. Currently, the market offer is based on atomized pure metals and pre-alloyed metal powders having particle sizes in the range of 10–45 μm, which must fulfill mandatory requirements such as the compliance to chemistry specifications (alloying elements and interstitials) and the ability to spread evenly across the machine’s build platform. All these properties can be controlled through different characterization methods, ranging from laser diffraction and scanning electron microscopy (particle size and morphology) to Hall and Carney funnels to assess the flowability, and X-ray diffraction (XRD), computed tomography (CT), and spectroscopic equipment to check the chemical composition and crystallinity [4,5,6,7].

When it comes to high-end application fields such as medicine and aerospace, and/or when the designed additively manufactured part is intended to be used for structural applications, a further crucial requirement, often underrated, is that the metal powder feedstock must be free from any foreign particulate contamination, such as light elements contamination (i.e., oxidation) and cross-contamination (exogenous), which is linked to the accidental mixing of metal powders having a different chemical composition [8,9]. Some combinations of metal powders’ cross-contamination were shown to have a detrimental effect on the mechanical properties, as reported by Brandão et al. [10] for Ti-6Al-4V and W, and by Gatto et al. [11] for maraging steel and metal oxides.

As suggested by Frazier [12], “alternatives to conventional qualification methods must be found based upon validated models, probabilistic methods and part similarities among others”, and while ASTM and other regulatory agencies are making a strong effort to develop new standards for additive manufacturing (AM), there is still a long way to go. New sets of standards will address the whole the manufacturing process chain, with a particular focus on critical applications (i.e., structural, aerospace, and biomedical).

Concerning the quality of the metal powder feedstock, the presence of a small amount (lower than 1 wt.%) of cross-contamination is not expected to significantly modify the rheological and macroscopic properties of the powder (granulometry, flowability, etc.), and cannot be identified with conventional laboratory XRD equipment. Therefore, the most commonly applied characterization techniques to check the morphology, dimensions, and inhomogeneities of metal powder feedstock are X-ray computed tomography (CT) and scanning electron microscopy (SEM) [4,13,14,15,16]. However, other characterization techniques such as electron backscattered diffraction (EBSD), secondary ions mass spectrometry (SIMS), and X-ray photoelectron spectroscopy (XPS) could be of high interest for cross-contamination detection and characterization. X-ray CT has proven to be extremely powerful for defects identification in AM parts generated by porosity [17] and/or cross-contamination [10,18]. On the other hand, automated systems such as computer-controlled SEM (CCSEM) equipped with software dedicated to powders identification and analysis in a particle-by-particle (PxP) approach [19] have a high potential for the determination of exogenous contamination detection and, therefore, feedstock qualification. Furthermore, image analysis software can be used to analyze results from CT scan and scanning electron microscopy micrographs [13]. SEMs are often equipped with energy-dispersive spectroscopy (EDS) detectors, which allow verification of the inhomogeneities in the chemical composition of the powders and easy identification and location of the cross-contamination in the sample [20].

Although the topic of spatter powders having different particle size than the virgin feedstock but the same chemical composition (besides an increased oxygen content) has already been addressed in the literature [21,22,23], the focus of this study is on the investigation of cross-contamination having a different chemical composition than the feedstock material, but comparable powder particle size.

Moreover, while many papers focus on the inspection of the powder feedstock to identify the contamination, there is a lack of experimental methods which can lead to a quantification of the contaminant. Therefore, the aim of the present paper was to develop an easy and reliable quantification procedure which is also effective for low cross-contamination concentrations (≤1 wt.%), based on a statistical approach which couples the SEM and EDS characterization techniques. The combination of the chemical information given by the backscattered electrons (BSE) signal in SEM and the EDS spectra and elemental maps was used to spot the contamination and then clearly quantify it. The experimental results indicate that by finely tuning the SEM–BSE working parameters, it is possible to enhance the quality of the acquired micrographs, suggesting a feasible implementation of the developed image-based quantification procedure in a machine learning environment.

## 2. Materials and Methods

The starting materials for the present study were two EOS (Electro Optical Systems GmbH, Krailling, Germany) virgin powders: (i) MaragingSteel MS1 [24], and (ii) Titanium Ti64 [25]. The first one is a maraging steel corresponding to US classification 18% Ni Maraging 300 and European 1.2709, while the latter is a Ti-6Al-4V alloy, corresponding to ISO 5832-3, ASTM F1472, and ASTM B348.

A controlled cross-contamination between the two powders was introduced in terms of weight percentage, so that samples of Ti-6Al-4V (Ti64) contaminated with maraging steel (MS) were prepared in 0.5, 1, and 2.5 wt.% concentrations, labelled Ti64 + 0.5MS, Ti64 + 1MS, and Ti64 + 2.5MS, respectively. The opposite mix was also evaluated, where the maraging steel powder was contaminated with the same amount in wt.% of Ti64 powder as mentioned above (namely MS + 0.5Ti64, MS + 1Ti64, MS + 2.5Ti64).

A small amount of powder was sampled according to ASTM F3049-14 (standard B215) and dispersed on an aluminum foil, to avoid further contaminations. The powder was then accurately spread with a spatula to prevent the superimposition of the powder particles, and it was attached on a scanning electron microscopy (SEM) stub using a graphite adhesive.

Scanning electron microscopy (SEM) observations were performed on a Zeiss Supra 40 field-emission SEM (Carl Zeiss Microscopy GmbH, Jena, Germany) equipped with a Bruker Z200 microanalysis (Bruker Nano GmbH, Berlin, Germany) for the energy-dispersive spectroscopy (EDS) inspections. Powders were accurately spread and attached on stubs for SEM; three stubs were characterized for each cross-contamination condition.

The chemical compositions of the pure and contaminated powders were checked by collecting three EDS spectra of each stub (three for each cross-contamination sample) on areas at the same low magnification (200×), using 20 keV accelerating voltage. A total area of 9 mm × 9 mm (ca.) occupied by powder coming from the same contaminated batch was investigated by quantitative EDS.

Further investigations were performed using the backscattered electrons signal and keeping the working parameters fixed for all the samples: (i) 60 µm aperture, (ii) 500× magnification, (iii) 8.3 mm working distance. Five micrographs for each stub were processed using the developed image analysis method, and the respective EDS elemental maps were acquired and used to confirm the presence and location of the contaminants. This procedure allows the investigation of a total area of 1.5 mm × 1.5 mm (ca.) for each powder mixture.

Concerning the accelerating voltage, a range of observations was carried out in order get an optimized balance between the EDS signal and the backscattered electrons contrast/brightness signal for SEM imaging.

This approach is advisable because BSE contrast depends on the chemical composition of the mixed materials and, for some combinations, the BSE signal could not generate a detectable contrast.

**Figure 1:**
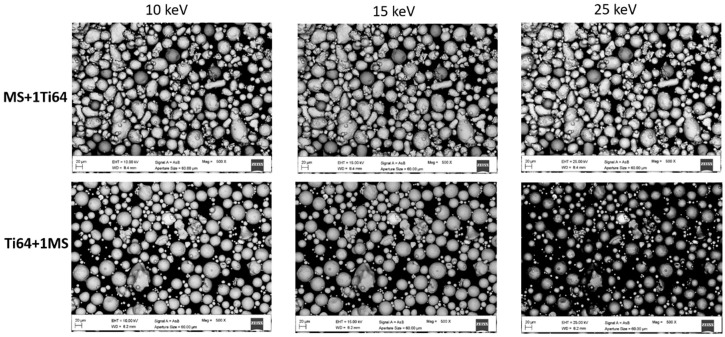
Comparison of micrographs taken at different values of accelerating voltage, for two cross-contamination samples, MS + 1Ti64 (upper row) and Ti64 + 1MS (lower row).

As can be seen in Figure 1, two powder mixtures (MS + 1T64 and Ti64 + 1MS) were taken as an example of the overall situation, and micrographs were acquired using the same SEM parameters except for the beam accelerating voltage which was varied from 10 to 25 keV. It is worth mentioning that no micrographs related to the 20 keV are shown, since during the contrast/brightness regulation the saturation of the BSE signal was reached before a clear image could be acquired. Therefore, with the current samples and the considered working parameters, the 20 keV which were used to acquire the chemical compositions were avoided during the rest of the measuring procedure. The most striking detail of Figure 1 is that while in the MS + 1Ti64 samples there is a clear contrast difference between the Ti-based particles (darker) and the Fe-based ones (brighter), the same cannot be said for the opposite samples (Ti64 + 1MS) at 10 keV. However, following the scanning electron microscopy theory [26], the BSE contrast should be enhanced by increasing accelerating voltage. This is confirmed by the 15 keV and even more by the 25 keV micrographs (Figure 1), but in the latter case the information linked to the particles’ morphology was lost and this lack of details makes the 25 keV condition unusable. Following this investigation, an accelerating voltage of 15 keV was selected for the observations of all the cross-contamination samples.

**Figure 2:**
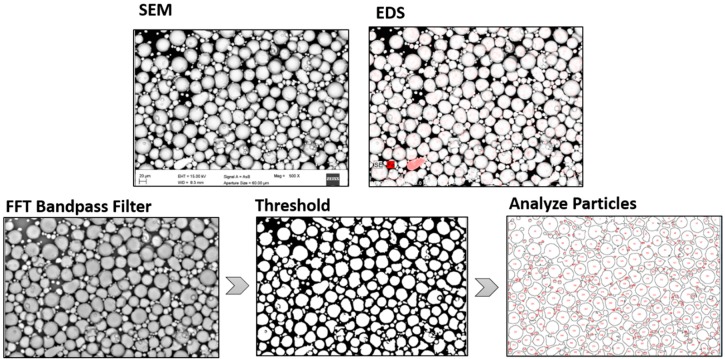
A graphical explanation of the procedure applied to the considered micrographs in order to highlight the cross-contamination particles and to measure them. FFT: fast Fourier transform.

Five micrographs were taken for each sample and then processed using the ImageJ software (version 1.51i, NIH, Bethesda, MD, USA) [27]; after the application of a fast Fourier transform (FFT) bandpass filter to binarize the micrographs, the “threshold” tool was used to isolate particles, whose details were then acquired using the “analyze particles” plugin. To easily spot the contaminants in the virgin feedstock, an EDS elemental map was acquired for each micrograph, and only the main element of the cross-contamination powder was mapped (Ti for Ti64 and Fe for MS). The full graphical procedure is reported in Figure 2.

To quantify the cross-contamination in each sample, the percentage ratio between the area occupied by the contamination particles and the virgin powder particles was calculated and labelled as “area ratio”, using only the information of the SEM–EDS system.

In order to show typical failure due to cross-contamination, specimens were built by direct metal laser sintering (DMLS), a powder bed fusion (PBF) technique equipped with an Yb (ytterbium) fibre laser system (EOSINT-M270, Electro Optical Systems GmbH, Krailling, Germany). DMLS ran under a protective inert gas atmosphere (oxygen lower than 1.5%) and the sintering parameters were set as follows: 200 W laser power, 0.2 mm diameter of the laser spot, 0.02 mm of layer thickness, scan speed up to 7 m/s, and building speed between 2 and 20 mm^3^/s. SEM observations and EDS analysis were used to characterize the fracture area of the contaminated samples.

## 3. Results and Discussion

**Figure 3:**
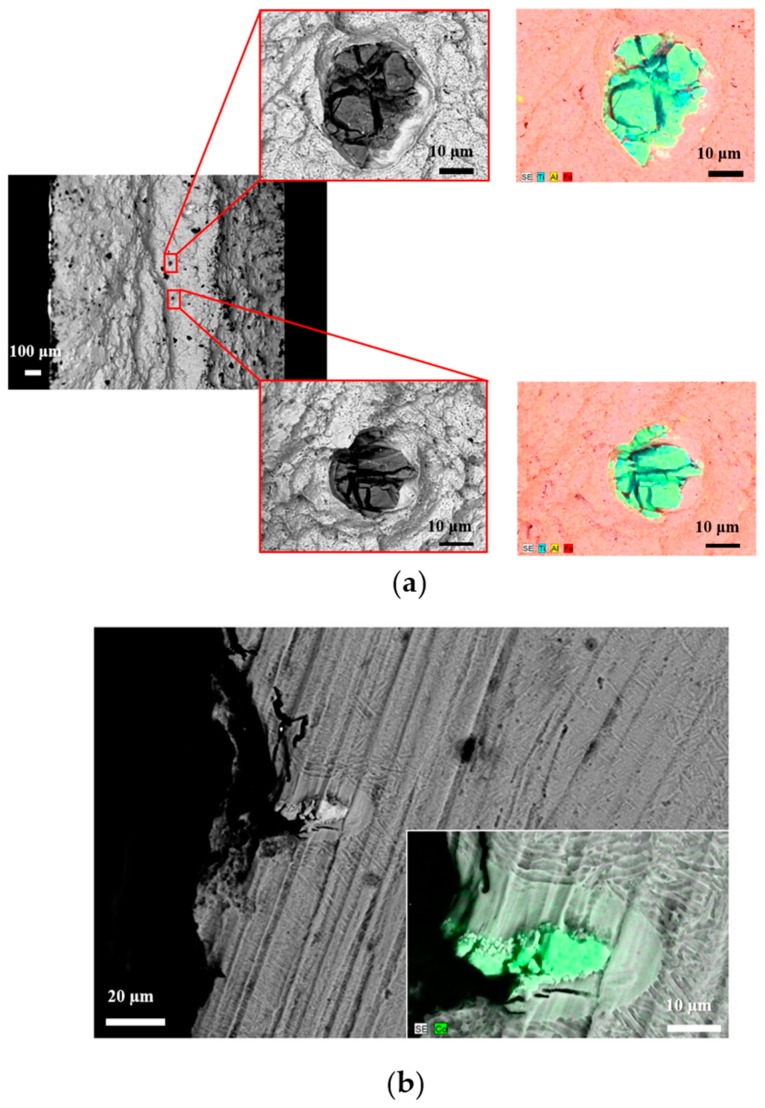
SEM micrographs of the fracture areas coupled with elemental maps of the cross-contamination particles for the (**a**) maraging steel sample with Ti64 inclusions, and (**b**) Ti-6Al-4V sample with Co-Cr-Mo inclusion.

The presence of cross-contamination can strongly affect the mechanical properties of additively manufactured metallic samples [10,11], since the discontinuities have thermal expansion properties different from the surrounding matrix. As an example, Figure 3 shows micrographs taken from unpublished results, highlighting defects in two contaminated samples.

Figure 3 reports the SEM micrographs of fracture surfaces in samples subjected to fatigue tests (Figure 3a) and flexural tests (Figure 3b), where cross-contamination inclusions were located in the failure zone. To enhance the difference between the metal matrix and the discontinuities, the backscattered electrons (BSE) signal was used in the image formation, so that the contrast was mainly given by the atomic weight of the elements. By coupling the SEM–BSE signal with elemental maps given by the energy dispersive spectroscopy (EDS) device, it was possible to fully characterize the contaminants.

In Figure 3a the inclusions had the typical compositions of another raw powder for additive manufacturing (namely, Ti64), while the discontinuity in Figure 3b had the typical composition of the Co-Cr-Mo AM raw powder. It must be stressed that in Figure 3b the contaminant particle was totally melted with the rest of the manufactured sample, and clearly acted as the crack’s generation site, being the weakest point of the part.

A crucial aspect that needs to be considered is the new perspective from where scientists and technicians need to look at failure analysis. Only by etching the Ti-6Al-4V alloy with the Keller’s etchant (95 mL H_2_O, 2.5 mL HNO_3_, 1.5 mL HCl, 1.0 mL HF) was it possible to highlight the presence of a cross-contamination element in the failure zone of Figure 3b, because the Co-Cr-Mo particle was perfectly mixed with the Ti64 matrix. The values of liquidus temperature for the two powders are quite close, being 1395 °C for the Co-Cr-Mo alloy and 1660 °C for Ti-6Al-4V; therefore, since the PBF system’s parameters were optimized to successfully melt the Ti64 particles, the first temperature was surely exceeded during the powder bed fusion process.

Figure 3 suggests that two types of cross-contamination can be classified in metal additive manufacturing. The first one can be named solidus–liquids and corresponds to the situation highlighted in Figure 3a, where the contaminant particles have a liquidus temperature higher than the metal matrix and therefore act as brittle unmelted discontinuities, such as those observed by Brandão et al. [10] and Gatto et al. [11]. The second type of cross-contamination can be named *liquidus–liquidus*, and matches the situation shown in Figure 3b, where the metal matrix and the contaminants were fused together because during the additive manufacturing process, the action of the high-energy beam raised the temperature above the liquidus level of the two alloys.

Both inclusions highlighted in Figure 3 show dimensions comparable with a single powder particle [28,29], suggesting the high degree of dependence between failure and the presence of such an impurity.

The compositions of all the powder samples were obtained by collecting EDS data from large areas (200× magnification) to get wt.% values representing the average situation in the sample. Deconvolution was used to resolve elements’ peaks overlap, and quantitative analysis was performed by the EDS software (ZAF correction [26]). Table 1 shows the results for the MS-based samples, where the wt.% of Ti and Al detected in contaminated samples were considerably higher than the nominal values. Moreover, these values increased as the wt.% of cross-contamination increased, showing a strong connection between the two, although not a direct proportionality.

The cobalt concentration was higher than the nominal value owing to the Fe secondary fluorescence linked to the proximity of their characteristic energy values, the Fe Kβ peak being at 7.06 keV while the Co Kα peak is at 6.92 keV.

Weight percentages of the main elements in Ti64 samples are reported in Table 2 and compared with the nominal composition.

Although the increment of iron content was consistent with the contamination introduced in the samples, the values obtained by collecting the signal from large areas were always lower than real contamination values (Table 2). The use of quantitative EDS analysis on large areas showed that cross-contamination could be highlighted quite reliably. This was also confirmed for the maraging steel samples, despite the remarkable amount of titanium which present in the nominal composition of the alloy. Note that for the sake of simplicity, only the main alloy elements are reported in Table 1 and Table 2.

**Figure 4:**
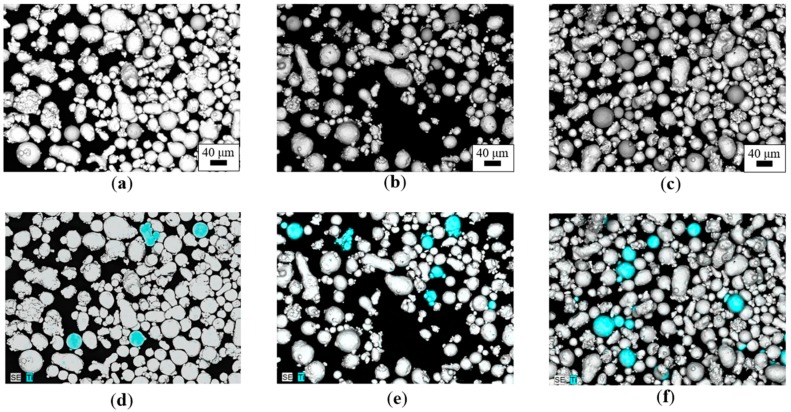
SEM–BSE micrographs of the MS-based cross-contamination samples: (**a**) MS + 0.5Ti64, (**b**) MS + 1Ti64, (**c**) MS + 2.5Ti64, and the respective energy-dispersive spectroscopy (EDS) elemental maps: (**d**) MS + 0.5Ti64, (**e**) MS + 1Ti64, (**f**) MS + 2.5Ti64. Blue particles correspond to Ti64.

**Figure 5:**
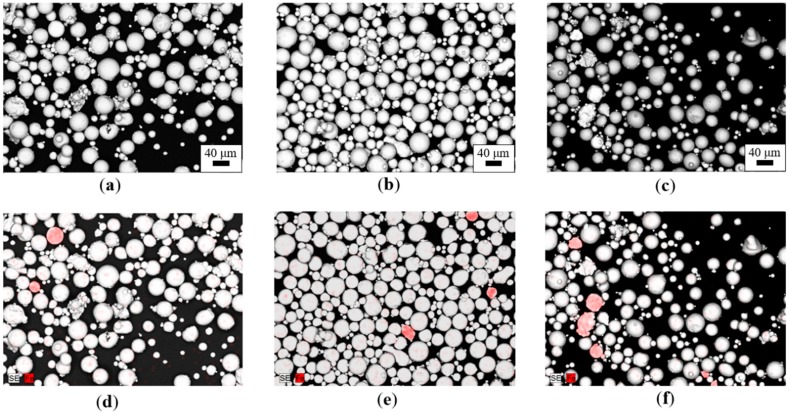
SEM–BSE micrographs of the Ti-based cross-contamination samples: (**a**) Ti64 + 0.5MS, (**b**) Ti64 + 1MS, (**c**) Ti64 + 2.5MS, and the respective EDS elemental maps: (**d**) Ti64 + 0.5MS, (**e**) Ti64 + 1MS, (**f**) Ti64 + 2.5MS. Red particles correspond to maraging steel.

Figure 4 shows typical SEM–BSE micrographs of the MS-based samples with different amounts of Ti64 cross-contamination, together with the respective EDS elemental maps (only Ti was mapped for the sake of simplicity), while representative micrographs and EDS maps of the Ti64-based samples are reported in Figure 5.

Note that in Figure 4 and Figure 5, despite the same amount of cross-contamination introduced in terms of weight percentage, the number of particles revealed by the EDS elemental maps in MS-based and Ti64-based samples were extremely different (much higher in the former condition). Figure 4 and Figure 5 suggest that density is the key to successfully detecting cross-contamination in metal powder feedstock for powder bed fusion. Indeed, despite the same amount of cross-contamination introduced in terms of weight percentage, the number of particles revealed by the EDS elemental maps in MS-based and Ti64-based samples were extremely different: This was due to the densities of the two powders: ρMS = 8.0–8.1 g/cm^3^ and ρTi64 = 4.41 g/cm^3^. Therefore, to reach the same weight percentage of cross-contamination, a larger number of Ti64 particles is necessary [30].

By applying the procedure shown in the experimental section, a comparative evaluation of the percentage ratio of the area occupied by the virgin and cross-contamination particles (namely, the “area ratio”) was calculated for all the samples, and the results are shown in Table 3.

The numerical values of the calculated area ratios in Table 3 show a direct proportionality with the amounts of introduced cross-contamination and, despite being extremely phenomenological, these area ratio values confirm the information arising from Figure 4 and Figure 5—that is, in order to reach the same amount of cross-contamination, a larger area should be occupied by the lightest contaminant powder. Moreover, the experimental results highlight that a comparative analysis of powder samples based on the combination of SEM–BSE micrographs and EDS elemental analysis is not only feasible, but was also characterized by an overall good level of reliability.

Following the detrimental effect of cross-contamination in powders for additive manufacturing reported in the literature [10,11] and the micrographs in Figure 3, a further study focusing on the determination of the link between the amount of cross-contamination in the powder feedstock and the mechanical properties of the additively manufactured parts is currently ongoing.

## 4. Conclusions

The presence of contaminants in metal powders for laser powder bed fusion is a technological challenge which is still limiting the use of this additive manufacturing technique. The present paper shows different possible effects of the presence of cross-contaminations in metal powder feedstock. Moreover, to verify and quantify the contamination, a combination of SEM–BSE observations, EDS analysis, and a subsequent image analysis procedure was used.

The main conclusions can be listed as follows:

- Two cross-contamination phenomena linked with the nature of the contaminant were observed and labelled, suggesting new perspectives for failure analysis procedures;
- By collecting EDS spectra on large areas of intentionally contaminated powder samples and comparing the elemental quantification with the nominal and pure powder compositions, the presence of the cross-contamination was detected;
- A fine tuning of SEM parameters allows optimization of the contrast information of the BSE micrographs and aids in obtaining reliable results with the EDS elemental mapping;
- When cross-contamination must be detected and measured, the density of the contaminant is the most relevant physical property;
- The use of the calculated area ratio between virgin powder particles and contaminants allowed numerical estimation of the cross-contamination. The experimental results showed a good agreement with the ratio of introduced cross-contamination amounts.

The results suggest the need to diversify material requirement in order to establish potential cross-contamination tolerance limits according to the targeted application. Because it is based on already well-established characterization techniques and reliable software, the implementation of the suggested image-based procedure in a machine learning environment could be the first step towards the next generation of quality standards of AM metal powder feedstock for high-end applications.

## Figures and Tables

**Table 1:** Comparison of wt.% concentrations (mean values and standard deviations) of major maraging steel (MS) elements (Ni, Co, Mo) and contaminants (Ti, Al).

Powders	Ni	Co	Mo	Ti	Al
MS (nominal)	17–19	8.5–9.5	4.5–5.2	0.6–0.8	0.05–0.15
MS pure	16.0 ± 0.3	10.7 ± 0.1	3.7 ± 0.2	0.9 ± 0.1	0.02 ± 0.01
MS + 0.5Ti64	15.4 ± 0.3	10.8 ± 0.1	3.5 ± 0.2	1.5 ± 0.2	0.05 ± 0.01
MS + 1Ti64	16.1 ± 0.2	10.4 ± 0.1	4.7 ± 0.5	4.0 ± 0.1	0.6 ± 0.3
MS + 2.5Ti64	15.6 ± 0.1	11.2 ± 0.1	4.9 ± 0.2	6 ± 1	0.7 ± 0.2

**Table 2:** Comparison of wt.% concentrations (mean values and standard deviations) of major Ti64 (Ti, Al) and contaminant (Fe) elements.

Powders	Al	V	Fe
Ti64 (nominal)	5.50–6.75	3.5–4.5	<0.30
Ti64 pure	5.9 ± 0.2	2.72 ± 0.03	ND ^1^
Ti64 + 0.5MS	5.4 ± 0.1	3.0 ± 0.1	ND ^1^
Ti64 + 1MS	5.9 ± 0.1	2.6 ± 0.1	0.8 ± 0.1
Ti64 + 2.5MS	5.3 ± 0.4	3.4 ± 0.1	1.3 ± 0.1

^1^ Not detectable.

**Table 3:** Mean values and standard deviations of the calculated area ratio for all the samples.

Sample Name	Area Ratio (%)
MS + 0.5Ti64	2.0 ± 0.2
MS + 1Ti64	3.6 ± 0.3
MS + 2.5Ti64	9.7 ± 0.6
Ti64 + 0.5MS	1.1 ± 0.4
Ti64 + 1MS	2.0 ± 0.2
Ti64 + 2.5MS	5.2 ± 0.2
